# Prediction and Characteristics of Angular Distortion in Multi-Layer Butt Welding

**DOI:** 10.3390/ma12091435

**Published:** 2019-05-02

**Authors:** Woo-Jae Seong

**Affiliations:** Industrial Application R&D Institute, Daewoo Shipbuilding & Marine Engineering Co., Ltd., 3370, Geoje-daero, Geoje-si, Gyeongsangnam-do 53302, Korea; wjseong@dsme.co.kr or wjseong@kaist.ac.kr; Tel.: +82-10-8838-6980

**Keywords:** multi-layer welding, systematic method, angular distortion, geometric principle, prediction curve, numerical approach

## Abstract

Multi-layer welding involves the process of stacking many beads, so it requires much time and effort to predict the deformation through experimentation or numerical analysis. In this study, a systematic method for predicting transverse angular distortion in multi-layer butt welding has been proposed. First, the database was established through bead-on-plate welding experiments, which consisted of the relationship between welding conditions, the bead geometry, the material thickness, and the angular distortion. Then, when the arbitrary welding conditions and the shape of the butt joint were input, the method calculated the angular distortion per pass using the geometric principle and the database. The obtained prediction curves were verified with the V-groove welding experimental results. In addition, the characteristics of angular distortion in multi-layer butt welding were discussed. It was found that the angular distortion curve is a function of the number of passes and groove geometry. This algorithm is based on a numerical approach that saves computational time using databases and geometry, so it is suitable for industrial applications.

## 1. Introduction

In industrial manufacturing processes, welding is used to assemble at least two members into a designed structure. Of many types of welding processes, arc welding provides high thermal energy to fuse metals to localized areas. For the consumable electrode method, the molten wire is transferred to the base metal and cooled, while the base material undergoes both the heating and cooling process by heat conduction from the molten wire. The molten wire shrinks by the amount of thermal strain, but is partially transformed into tensile plastic strain by the amount that is not contracted due to the adjacent constraints. In the case of the base material, the amount of compressive plastic strain generated during heating is larger than that of tensile plastic strain during cooling, leading to permanent deformation. Therefore, since the material is not completely shrunk, tensile stress remains with a wider lattice spacing. By stress equilibrium, the surrounding region has compressive residual stress [1,2,3,4,5]. It has been well known that tensile residual stress affects brittle fracture, fatigue life, stress corrosion cracking, weld cracks, and buckling strength [6].

The welding distortion produces geometrical misalignments and gaps between structures, making it difficult to assemble and requiring additional work. It degrades the product quality and productivity. The structural members such as legs, nodes, main truss, and girders in the offshore platform and hull girders in the ship are designed to withstand heavy loads. Since they are almost thick plates, multi-layer welding is applied during fabrication. In particular, under compressive loading, the initial distortion induced by welding may act as a geometrical imperfection to buckling. It has been generally recognized that the initial deflection reduces the buckling strength. Timoshenko [7] explained that the initial curvature on deflection lowers the buckling strength, and its curve is similar to that of the secant formula by eccentric loading. Therefore, it is necessary to predict and control the welding distortion more accurately. The rules and standards of DNV GL [8], which is an international accredited registrar and classification society, also specify tolerances of alignment and straightness for structural design based on this theory. In the shipbuilding and heavy industries, product design and fabrication are carried out in accordance with these types of standards to prevent severe disasters due to buckling instability. In addition to conventional industries, it has been reported that thermal deformation problems arise during multi-layer deposition in the additive manufacturing (AM) process. Mukherjee et al. [9] mentioned that the thermal deformation causes dimensional inaccuracy in AM parts. They derived a predictive equation from the measured strain data and suggested that the thermal strain can be reduced by adjusting the variables such as the laser power, layer thickness, and scanning speed. Xie et al. [10] pointed out that the constraining force is the most influential factor for the thermal deformation and related to material properties, temperature, and cross-sectional area of the layer. They observed that the displacement increases proportionally as the layers are deposited.

The angular distortion of one-pass welding is caused by non-uniform transverse shrinkage in the thickness direction [6]. The distortion is susceptible to the amount of weld deposition, temperature gradient through the thickness, and constraint. Through the laser welding experiment and their theoretical model, Xie et al. [11] revealed that the direction and size of the angular distortion are dependent on the asymmetry of the cross-sectional profile of the bead. The bead deposition of the cross-section can change with various processes and conditions. Besides, the angle distortion can be affected by the properties of the weld material, the distribution of the plastic strain, and even pores. The temperature distribution is governed by the combination of the flow of molten fluid deposited on the groove joint, the cooling conditions by radiation and convection, and the thermal properties of the materials. Deng and Liang [12] emphasized that the temperature gradient strongly dominates the angular distortion through the fillet welding simulation. Satoh and Terasaki [13] also stated that the angular distortion varies with the initial temperature, which influences the temperature gradient along the thickness. Sepe et al. [14] evaluated the effect of the pre-heating and post-heating treatments on temperature distribution, the residual stress, and distortion. Materials lose stiffness at an elevated temperature, so the distortion is determined by the surrounding constraints such as the initial setting and fit-up conditions.

There have been many fundamental studies on welding distortion under various welding conditions and materials. Okerblom [15] summarized through bead-on-plate welding experiments that the angular distortion has a linear relation with the ratio of the heat input (*H*) to the square of the thickness (*t*) when the penetration is less than 0.6-times the thickness. When the penetration increases further, the non-fusion zone under the fusion zone easily develops into plasticity due to the temperature rise, and it becomes difficult for it to act as the rotation point for bending. Satoh and Terasaki [13] investigated the effect of the heat input, the plate thickness, and various materials on the deformation through vast experiments. Furthermore, they deduced formulae to predict welding distortion based on heat flow and thermal elastoplastic theory. The results revealed that welding distortion depends on $H/t^{2}$ (the heat input divided by the square of the thickness) with the same material. In the case of mild steel, the distortion is proportional to $H/t^{2}$ regardless of welding processes when the $H/t^{2}$ is less than 2500 cal/cm${}^{3}$. Rui et al. [16] presented the elastic model based on the inherent strain method from the thermal elastoplastic analysis and experiments. Their database showed that the transverse angular distortion increases until $H/t^{2}$ arrives at 10 kJ/mm${}^{3}$ and then decreases until $H/t^{2}$ reaches 40 kJ/mm${}^{3}$. The results of the above studies are in common in that the distortion is a function of the heat input and thickness. Specifically, the angular distortion is proportional to the heat input and inversely to the square of the thickness, then decreases after the maximum value.

In recent years, new approaches have been addressed to identify these relationships. Tian et al. [17] adopted the back-propagation neural network (BPN) model to estimate the angular distortion and the shrinkage in the bead-on-plate welding with S304 stainless steel. Various welding speeds, electric currents, and voltages with a constant thickness were used as input parameters. They found that angular distortion increases up to the heat input of 1 kJ/mm, then decreases with the further increase in heat input. Mochizuki and Okano [18] explored the relationship between angular distortion and the mechanical melting region where the material significantly loses strength and stiffness. They concluded that the angular distortion increases linearly with the ratio of the mechanical melting region to the square of the thickness up to the threshold, then decreases. The ratio at the maximum distortion appears to be between 0.6 and 0.8. Additionally, It was found that the effect of bead reinforcement on the deformation was negligible, especially for large thickness.

Multi-layer or multi-pass welding is a process in which several welding passes create one layer and several layers are filled in the weld joint. A new layer is deposited on top of the previous layer, so the effect of the previous layer on the welding deformation should be examined. Luo et al. [2] analyzed the mechanism of the inherent strain considering the existing weld bead in multi-layer welding. The results of the experiment and the thermal-elastoplastic analysis showed that the residual stress induced by the preceding weld layer reduces the angular distortion at the current pass under the condition of the low heat input and high constraint. Okano et al. [19] observed through the X-groove welding experiment and the numerical analysis that the residual stress by the previous welding pass changes the mechanical melting temperature and plastic strain, so the angular distortion decreases. In the field of plate forming with thermo-mechanical behavior similar to welding, it has been reported that the deformation per pass in multi-pass heating decreases as the pass progresses. Vega et al. [20] unveiled through simulation that the residual stress generated by line heating affects the deformation of the next line heating. Sprenger et al. [21] asserted in laser forming that one of the causes is the strain hardening due to dislocation entanglement induced in the previous pass.

Since the groove cut between plates is filled with the molten wire, the number of passes is determined by the amount of the deposited metal per length. Research on the wire melting rate has also been performed based on arc welding physics. Murugan and Gunaraj [22] predicted bead dimensions through a mathematical model in submerged arc welding (SAW) of pipes. They found that the wire feed rate and the arc voltage enlarge the heat-affected zone (HAZ), but the welding travel speed and the nozzle-to-plate-distance reduce the bead area. Furthermore, the wire feed rate increases the bead size, while the arc voltage increases bead width, but decreases the penetration and reinforcement of the bead. Khanna and Maheshwari [23] made a prediction model for weld bead size in gas metal arc welding (GMAW) of stainless steel 409 M using the technique of central composite rotatable design. Their finding was that the welding current increases the penetration, width, reinforcement, and dilution, but the welding speed lowers all bead dimensions, while the arc voltage increases all the bead dimensions except for the reinforcement. The cases of flux cored arc welding (FCAW) were also consistent with the results of GMAW and SAW. Sadek et al. [24] investigated the effect of welding variables on the bead geometry and HAZ width. They observed that the cross-sectional area of weld metal and HAZ width decreases with increasing the welding travel speed. Palani and Murugan [25] developed a regression model using response surface methodology to estimate the weld geometry for FCAW cladding of 317L. It was seen that the welding current increases the penetration depth, the bead size, and the penetration area. These studies support the fact that the electric current plays a dominant role in the bead size because the electrode wire is melted by electrical resistance heat [26,27]. Boccarusso et al. [28] studied the relationship between bead morphology and fatigue life with various welding process parameters. They concluded that the heat input changes the bead aspect ratio between the maximum height and width of the joint, which influences fatigue life.

There has been research on the systematic approach to the prediction of welding distortion for practical applications. Kim et al. [29] obtained a predictive equation of angular distortion in multi-layer welding considering the heat input and the effective bending rigidity of each welding pass. They provided the proper welding sequence for the double-sided welding of stainless 316LN in a vacuum and a cryostat vessel. Murugan and Gunaraj [22] presented the mathematical model for estimating angular distortion in GMAW. Process parameters were used to figure out the effects on the distortion, which were a function of time between successive passes, the number of passes, and wire feed rate. It was proven that the number of passes influences the angular distortion and that the others do not. Ha and Choi [30] developed a strain-boundary method based on the FE-shell element and introduced an imaginary temperature pair considering the weld joint geometry. A predictive curve of cumulative angular distortion was extracted to solve practical engineering problems. Given the current deposition amount, the final angle can be acquired without time-consuming simulation. Adamczuk et al. [31] suggested a prediction methodology of angular distortion in multi-layer butt welding. Three analytical equations were defined through the V-groove welding experiment with different heat inputs. The model gave the cumulative angles with the different heat input and welding pass. It was concluded that the angular distortion reaches the maximum at the fourth pass and then decreases with further passes regardless of heat input.

This study aims to develop a systematical method to predict angular distortion in multi-layer welding. Through the bead-on-plate welding experiment, the relationship between heat input, bead cross-section, angular distortion, and thickness was defined as the deformation-related properties, and two databases were constructed. Then, an algorithm was proposed by a numerical approach using geometric principles and the databases.

## 2. Materials and Methods

### 2.1. Experimental Study for the Database

As the welding pass progresses, the welding deformation continues to increase. Since the thickness increases by the height of the accumulating layer, the angular distortion per pass gradually decreases. On the other hand, the amount of weld deposit per pass may vary depending on the heat input. Heat input and welding geometry determine the total number of passes, which can affect angular distortion. Therefore, the main factors for angular distortion in multi-layer welding can be narrowed down to weld conditions, thickness, and joint geometry.

**Figure 1:**
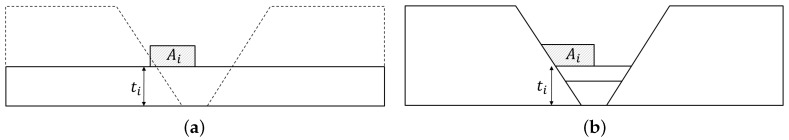
Assumption of angular distortion under multi-layer welding, $t_{i}$: current thickness, $A_{i}$: cross-sectional area of the current bead: (**a**) bead-on-plate welding. (**b**) V-groove butt welding.

The ideal prediction method is required to output the angular distortion when the user inputs information about arbitrary welding conditions and joint shape. Thickness data should be updated automatically during computation. To this end, this study clarified the relationship between these variables through bead-on-plate welding experiments. This was based on the assumption that the angular distortion in the bead-on-plate welding and the V-groove welding is equivalent under the same thickness and heat input, as shown in Figure 1a,b. In reality, the difference in thickness at the location away from the weld joint can affect the distortion, but the bending moment due to the weld shrinkage and cross-section may be much more dominant. The verification of this assumption is dealt with in Section 3.1. The materials and methods for the bead-on-plate welding experiments are described as follows.

#### 2.1.1. Materials

The size of the specimen was 1500 × 1000 mm with different thicknesses. The width was chosen to minimize the longitudinal distortion along the weld line. Low carbon steel AH32, which is mainly used for ship structures, was adopted as the base material. FCAW with a the wire diameter of 1.2 mm and 100% CO${}_{2}$ shielding gas was applied. The type of the wire was E81T1-K2C, conforming to the specification of American Welding Society [32]. The chemical compositions and mechanical properties of the base and the welding material are summarized as Table 1.

#### 2.1.2. Bead-on-Plate Welding Experiment

**Figure 2:**
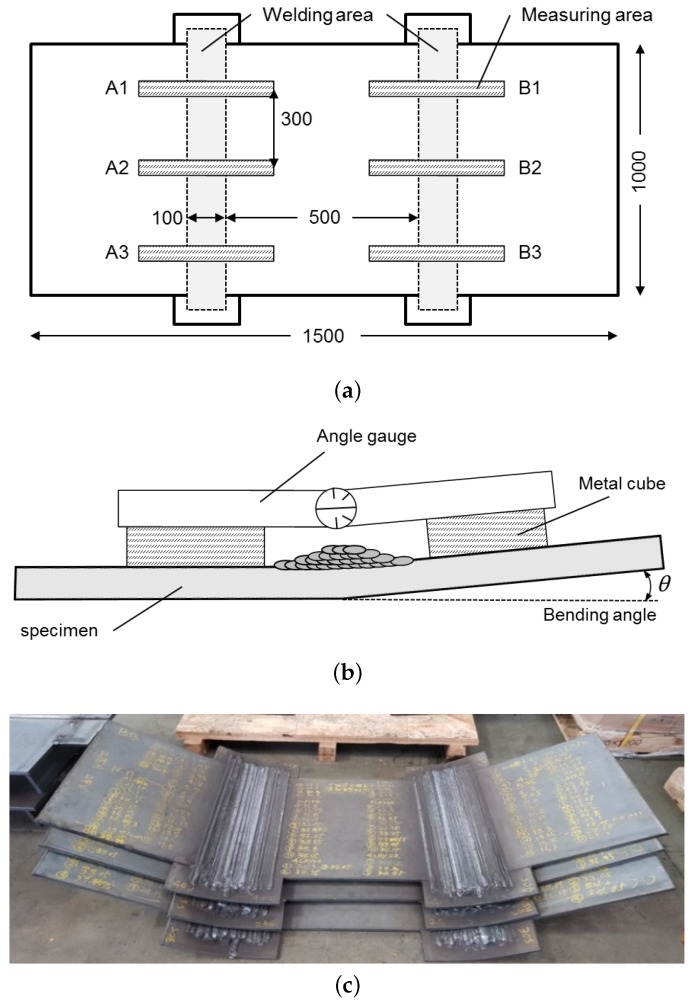
Bead-on-plate experiment: (**a**) information on welding and measurement; (**b**) measurement of angular distortion; (**c**) welded specimens.

The bead-on-plate welding was conducted at two welding areas where the width of the plate was separated into three equal parts, as shown in Figure 2a. All passes and layers were subjected to the same welding condition for one welding area. By varying the welding speed at constant current and voltage, different heat inputs were acquired to each welding area. Both edges were free to bend. During the multi-pass welding, the plate was completely cooled down before the next pass welding, and the angular distortion was measured for each welding pass. The measurement points were chosen at three points (A1, A2, A3 or B1, B2, B3) to avoid the transient temperature near the front and the end of the specimen. The average of the measured values was adopted as the bending angle (angular distortion). Figure 2b shows the angle measurement with a digital angle gauge on flat metal cubes. The size of the cube was 150 × 50 × 50 mm. The welded specimens with multi-layer welding are shown in Figure 2c. Specimens of 12 mm in thickness were welded to four layers, while the others were welded to two layers. The degree of angular distortion determined the accuracy of the measurement, so the number of passes for each layer varied with the amount of deformation. For less distortion, more passes were required. Table 2 shows the experimental conditions. Since the experiment was repeated twice, a total of 22 specimens were used for 22 conditions.

### 2.2. Databases for Algorithm

#### 2.2.1. Database #1: Bead Area with Current-Velocity Parameter

In the arc welding, electric energy is transformed into heat energy sufficient to fuse the metal. In the case of FCAW, the electrode wire is melted by resistance heat of the wire extension plus arc heat. The wire melting rate is a function of quadratic current as written in Equation (1) [26,27].

(1) $$M^{\prime }=a_{1}I+a_{2}I^{2}$$

where $M^{\prime }$ is the wire melting rate (kg/s), $a_{1}$ is the constant of proportionality for anode or cathode heating, $a_{2}$ is the constant of proportionality for electric resistance heating, and *I* is the electric current (A). The first and second terms stand for the arc heat and resistant heat. The cross-sectional area of the bead can be obtained by dividing the melting rate by the welding travel speed and the wire density, as expressed in Equation (2).

(2) $$A_{b}=\frac{M^{\prime }}{\rho v}=\frac{a_{1}}{\rho v}I+\frac{a_{2}}{\rho v}I^{2}≃\frac{C_{1}I^{2}}{v}+C_{2}$$

where $A_{b}$ is the cross-sectional area of the bead (mm${}^{2}$), ρ is the wire density (kg/mm${}^{3}$), *v* is the welding travel speed (mm/s), $I^{2}/v$ is the current-velocity parameter (A${}^{2}$s/mm), and $C_{1}$ and $C_{2}$ are constants.

**Figure 3:**
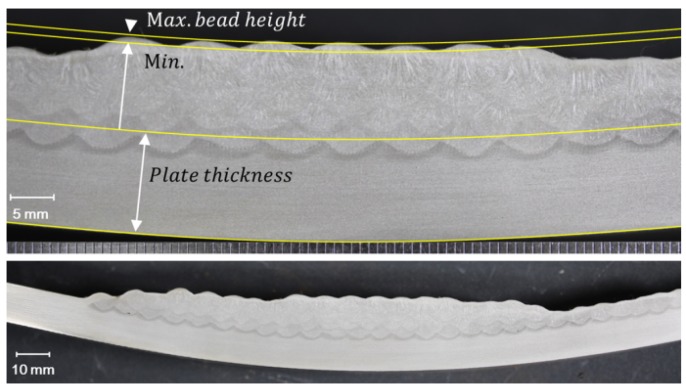
Cross-section of multi-layer beads; specimen thickness of 12 mm, 29 V, 285 A, 40 cm/min (1.0 kJ/mm).

The density of the wire material had a constant value, and the square of the current was much larger than the current, so the cross-sectional area of the bead was almost proportional to the square of the electric current and inversely proportional to the welding travel speed. In this study, the square of the electric current divided by the welding travel speed was defined as the current-velocity parameter ($I^{2}/v$). In order to define the unknown constants, heating conditions and the cross-sectional area of the bead were acquired. After cutting welded specimens, the total area of the multi-beads was measured by pixel values, and the area per pass was obtained. Figure 3 shows an example of the macro section of the multi-layer welded specimen. Since the surface of the bead had a wave shape, the average of the maximum and minimum height was selected as the total height of the layers. Then, it was divided by the number of layers for the bead height per layer

**Figure 4:**
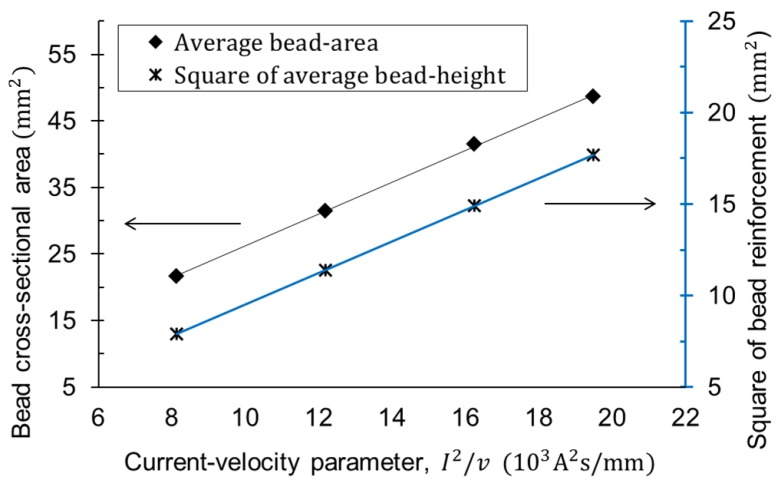
Database #1: relationship between current-velocity parameter and bead cross-sectional shape.

Figure 4 shows the result that the cross-sectional area of the bead had a linear relation with the current-velocity parameter, which is agreeable with Equation (2). This result is a property required to predict the welding deformation and was named as Database #1. If the heat resistance is different, that is, if the material or shape of the wire is changed or if a different welding process is used, a new database should be built up through the bead-on-plate welding experiment in the same way as described above. The following Equation (3) includes the determined constants.

(3) $$A_{b}=\frac{C_{1}I^{2}}{v}+C_{2}$$

where $C_{1}=2.3954\times 10^{-3},C_{2}=2.2536$, and $I^{2}/v$ is the current-velocity parameter.

Figure 4 also contains the result of the bead height (reinforcement) with the current-velocity parameter, as well. The square of the height was employed to equal the unit of area. The bead reinforcement was used as the reference height of a layer in the proposed algorithm and denoted as Equation (4).

(4) $$t_{ref}=\sqrt{\frac{C_{3}I^{2}}{v}+C_{4}}$$

where $C_{3}=8.5862\times 10^{-4},C_{4}=0.9356$.

#### 2.2.2. Database #2: Angular Distortion with Heat Input-Thickness Parameter

**Figure 5:**
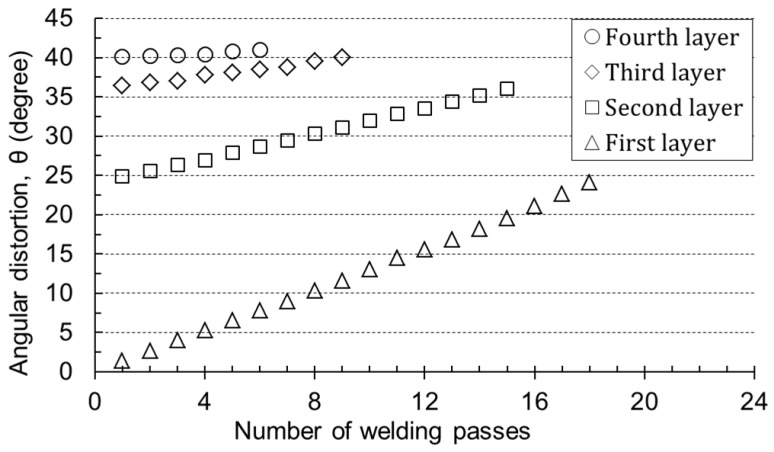
Measured angular distortion with the number of welding passes; specimen thickness of 12 mm, 29 V, 285 A, 40 cm/min (1.0 kJ/mm).

Figure 5 shows the angular distortion regarding the number of passes under the same thickness and heat input. It was observed that the angular distortion increased proportionally with the number of passes on the same layer, but for the slope, the angle per pass decreased with the number of layers. For example, the change of the angular distortion in the second layer was less than that in the first layer because the thickness was added by the bead height of the first layer.

**Figure 6:**
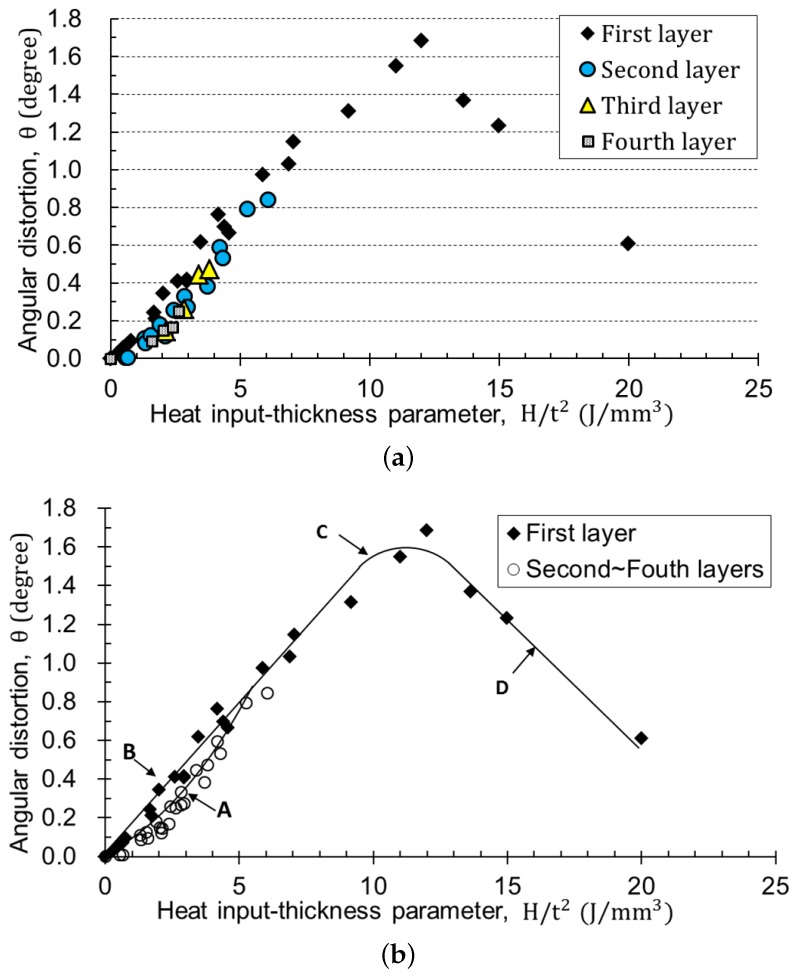
Database #2: relationship between angular distortion and heat input-thickness parameters where thermal efficiency is 0.8: (**a**) with different layers; (**b**) regression curves for calculation.

On the other hand, the measured angular distortions under different welding conditions are displayed in Figure 6. The heat input (*H*) shown on the horizontal axis is defined as the product of the thermal efficiency, the electric current, the voltage, and the inverse of the welding travel speed, which means the effective thermal energy supplied to the base material per unit length. The thermal efficiency is a value that considers the energy lost in the process of converting electrical energy into thermal energy and transferring heat to the parent material. It depends on the welding process and is generally obtained by comparing the experiment with numerical analysis. This study used 0.8 as the thermal efficiency of the FCAW process. The heat input divided by the square of the thickness was defined as the heat input-thickness parameter ($H/t^{2}$).

Figure 6a shows the angular distortion with the heat input-thickness parameter. The data marked as the first layer represents the angular distortion by welding directly onto the plate without any previous bead. The second layer was welded over the first layer, so there were previous beads. The fourth layer data symbolize the angular distortion when a layer was additionally welded over three layers. In the case of the first layer welding, the angular distortion rose linearly with the heat input-thickness parameter, reached the maximum at about 12 kJ/mm${}^{3}$, and then decreased. The trend of the results agreed well with previous studies [13,15,16].

As shown in Figure 6b, the angular distortion of the second to the fourth layer was less than that of the first layer when the heat input-thickness parameter ranged from 1–6 kJ/mm${}^{3}$. The maximum gap was 56% at 2 kJ/mm${}^{3}$. This implies that the previous layer had an effect on the decrease in the angular distortion at the present pass. Possible causes are the tensile residual stress and the strain hardening induced by the previous layer and the dilution with the wire material [2,19,20,21]. In principle, tensile residual stress cut down a portion of the compressive stress by heating, and the strain hardening increased yield strength due to dislocation entanglement. Therefore, the amount of the plastic deformation was reduced as a whole. This aspect gradually diminished when the heat input-thickness parameter was out of the range of 1–6 kJ/mm${}^{3}$. The angular distortion began to decrease over about 12 kJ/mm${}^{3}$. This indicates that the temperature gradient through the thickness became mild because the ratio of heat input to the thickness increased.

The result as shown in Figure 6b was the property affecting the welding deformation and was named as Database #2. Four curves were generated from the measured data by the least squares method. The lines B and D are the first-order, and the curves A and C represent the second-order. The curve A corresponds to the data considering the previous weld layer, and the others do not. The curves B, C, D, and A are summarized as Equations (5)–(8), respectively.

(5) $$\theta =C_{5}\frac{H}{t^{2}}$$

where $C_{5}=0.155$ ($0\leq H/t^{2}<9.3345$), *H* is the heat input, *t* is the thickness, and $H/t^{2}$ is the heat input-thickness parameter:

(6) $$\theta =C_{6}{\left(\frac{H}{t^{2}}\right)}^{2}+C_{7}\frac{H}{t^{2}}+C_{8}$$

where $C_{6}=-0.0243$, $C_{7}=0.5518$, $C_{8}=-1.5866$ ($9.3345\leq H/t^{2}<13.7265$),

(7) $$\theta =C_{9}\frac{H}{t^{2}}+C_{10}$$

where $C_{9}=0.13$, $C_{10}=3.1936$ ($13.7265\leq H/t^{2}<20.0$),

(8) $$\theta =C_{11}{\left(\frac{H}{t^{2}}\right)}^{2}+C_{12}\frac{H}{t^{2}}+C_{13}$$

where $C_{11}=0.0131$, $C_{12}=0.07848$ ($0\leq H/t^{2}<6.3174$).

### 2.3. Algorithm for the Prediction of Angular Distortion

**Figure 7:**
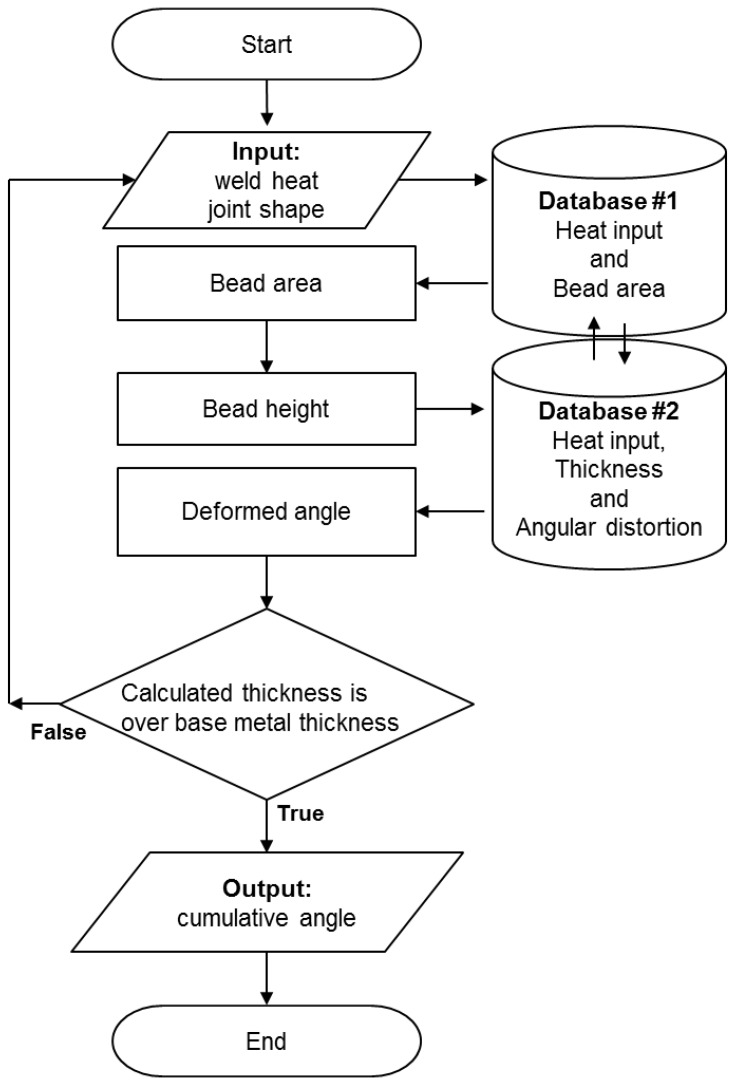
Flowchart of the prediction for cumulative angular distortion.

The database described above represents the correlation between welding conditions, bead geometry, thickness, and angular distortion. In order to connect the database with various weld joints, the joint geometry should also be considered. The proposed method uses both the database and geometric principle so that the angular distortion can be predicted under arbitrary weld joint and welding conditions. The flowchart of the algorithm is summed up in Figure 7, and the detailed procedures are as follows.

**Figure 8:**
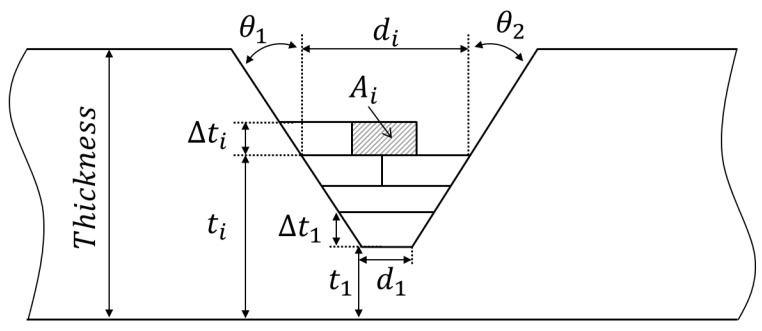
Geometrical definition of multi-layer butt welding.

1. Input information on weld joint geometry and the welding conditions in the current layer.
  Weld joint geometry consists of bevel angles, the thickness, the root gap, the root face, and the thickness below the weld root. The welding condition is the average of all passes in the current layer.
2. Calculate the cross-sectional area of the bead using Database #1 and the input data of Step 1.
  Database #1 is composed of the relationship between the current-velocity parameter and the bead area.
3. Calculate the beat height from the bead area and the weld joint geometry.
  As shown in Figure 8, every layer has a trapezoidal shape in the case of the V-groove joint. Geometrically, the height of the trapezoid can be calculated with the bead area ($A_{i}$), bevel angles ($\theta_{1}$, $\theta_{2}$), and the bottom length of the bead ($d_{i}$). The subscript *i* denotes the layer number, and *j* is for the bead number in the corresponding layer. The layer height ($\Delta t_{i}$) was updated as *j* was incremented one by one in the current layer *i*. The calculation was repeated until $\Delta t_{i}$ was greater than the reference height $t_{ref}$ in Equation (4). At the time of termination, $\Delta t_{i}$ was determined as the bead height of the *i*th layer as written in Equation (9), and the value *j* was stored as the total number of beads in this layer.
  (9) $$\Delta t_{i}=\frac{-d_{i}+\sqrt{d_{i}^{2}+2j\Delta A_{i}\left(\tan\theta_{1}+\tan\theta_{2}\right)}}{\tan\theta_{1}+\tan\theta_{2}}$$
  The current thickness plus the bead height will be used as the thickness for the calculation of the next layer, which is written as Equation (10).
  (10) $$t_{i+1}=t_{i}+\Delta t_{i}$$
4. Calculate the top length of the layer:
  The top length ($d_{i+1}$) can be obtained by Equation (11) with the bottom length ($d_{i}$) and the height of the current layer ($\Delta t_{i}$). The top length is used as the bottom length for the calculation of the next layer.
  (11) $$d_{i+1}=d_{i}+\Delta d_{i}$$
5. Calculate the angular distortion at the current pass:
  In the current layer *i*, the angles from Bead Number 1 – *j* are extracted through Database #2 using the heat input and thickness ($t_{i}$). The data $h_{i}$ were the thickness calculated in the previous layer. Database #2 was made up of the relationship between the heat input, the thickness, and the angular distortion. When the heat input-thickness parameter was less than 0 or greater than 25 kJ/mm${}^{3}$, the output of the angular distortion was zero.
6. The layer number *i* increased by one, and Steps 1–5 above were repeated.
  The value *j* was initialized to one before each layer calculation. The calculation terminated when $t_{i}$ was greater than the thickness of the base material.
7. Calculate the accumulated angular distortion by summing the angles produced for each pass.

## 3. Results and Discussions

### 3.1. Algorithm Validation through Experiment

**Figure 9:**
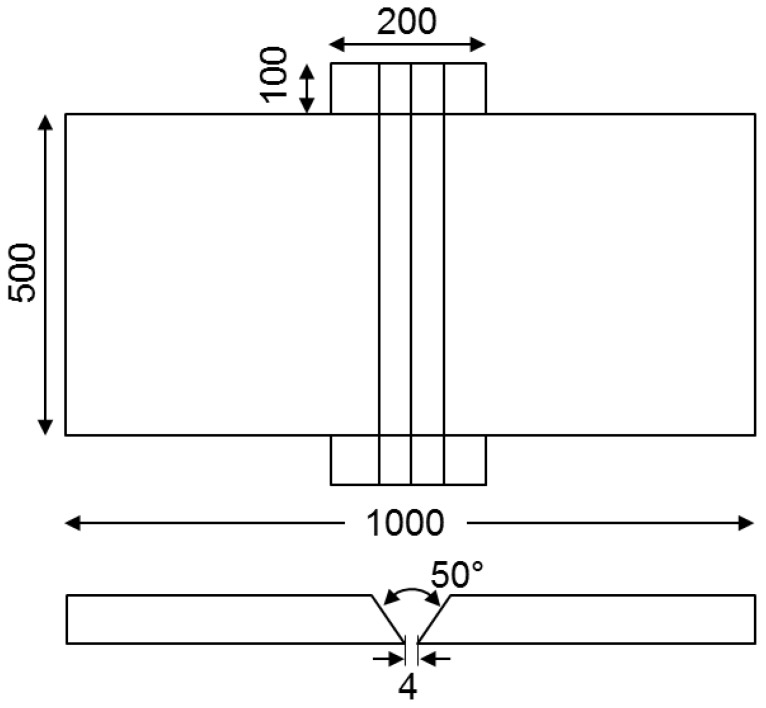
Specimen of the V-groove multi-layer welding for algorithm validation; root gap: 4 mm, root face: 0 mm, thickness: 28 mm.

The multi-layer welding experiments with the V-groove were conducted to verify the proposed prediction method. Figure 9 shows a test specimen of size 1000 × 500 mm. The welding conditions are included in Table 3, and the other conditions were the same as those in the database experiment.

**Figure 10:**
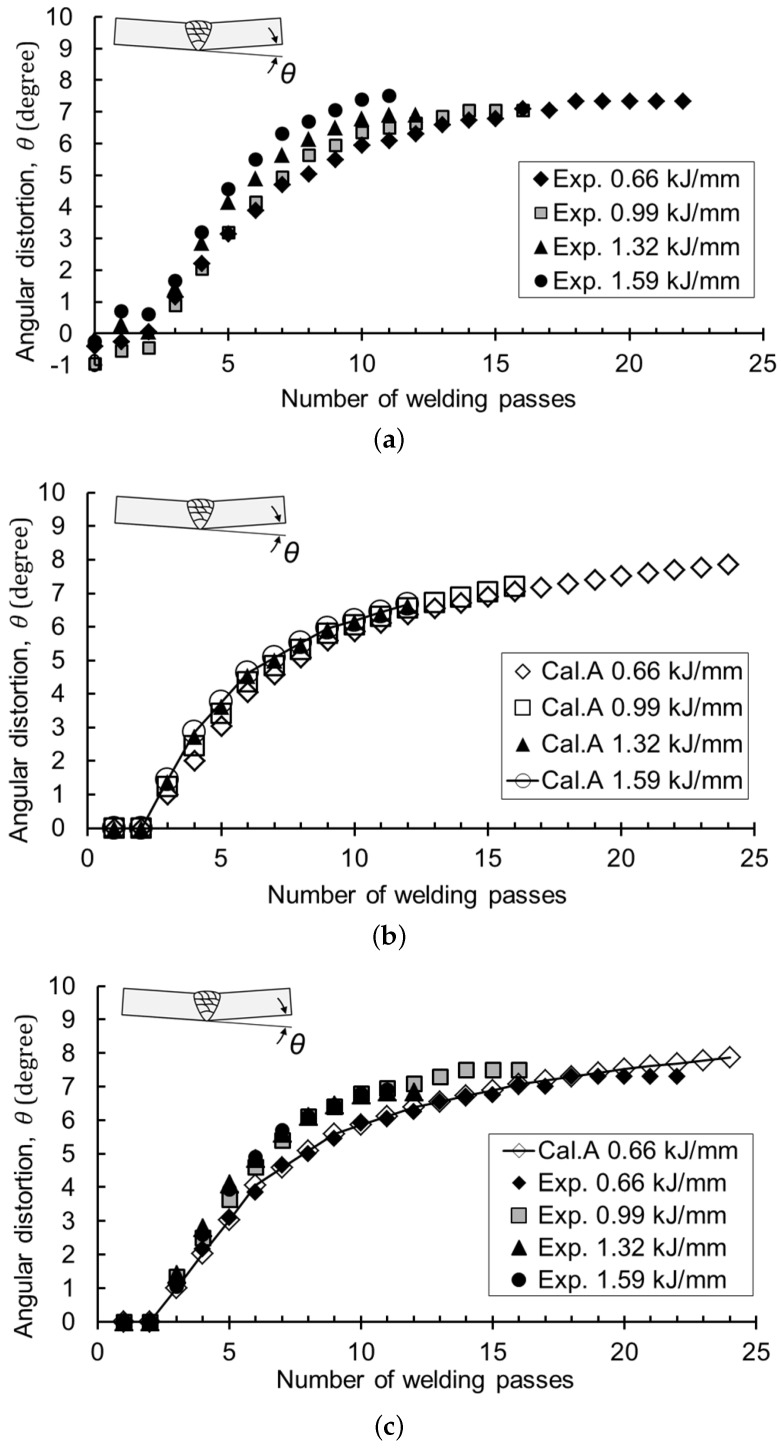
Result of angular distortion in V-groove multi-layer welding: (**a**) experimental (Exp.) results; (**b**) calculation (Cal.) results; (**c**) comparison of the experiment with calculation.

Figure 10 shows the results of the angular distortion with the number of welding passes. In the legend, “Exp.” and “Cal.” stand for the data by experiment and calculation, respectively. Pass Number 0 had the initial distortion as shown in Figure 10a, which was due to the fit-up condition before welding. Then, all the angular distortions slightly increased at the first pass. After the second pass, the distortion became higher at the low heat input and lower at the high heat input.

Generally, in butt welding, there is no leverage to cause bending because there is no previous bead in the first layer. For the second layer welding, the first layer beads can act as a lever. However, since the height of the first layer is not large enough, most of the first layer beads are thermally affected. For most first and second layers, the heat input-thickness parameter was much higher than 25 kJ/mm${}^{3}$, which produced small angular distortion, as shown in Figure 10a. The value can be negative, which means the reverse distortion. Furthermore, the angular distortion can be zero if the shrinkage is uniform through the thickness. In this case, the shrinkage has the maximum instead. In other words, the deformation up to the second pass was sensitive to the amount of weld deposition, the temperature gradient through the thickness, and the surrounding constraint [11,12,13,14]. These phenomena can be deduced from Figure 6a by extrapolating the data. From the third pass, the parameter became lower than 25 kJ/mm${}^{3}$, and the distortion pattern became apparent to achieve accurate prediction.

For this reason, when the heat input-thickness parameter was over 25 kJ/mm${}^{3}$, the algorithm initialized the corresponding angular distortion to zero. Figure 10b shows the calculation of normalizing the angular distortion to zero at the first and second pass. “Cal. A” represents the calculation using Curve A in Database #2, as shown in Figure 6, which considers the effect of the previous pass. The calculation shows that the angular distortion was slightly larger at higher heat input, but the amount was so small that all results can be regarded as following a single curve.

All of the experimental data below were calibrated so that the angle of the second pass was zero for quantitative comparison with the calculation results. This study does not cover the range where the heat input-thickness parameter was greater than 25 kJ/mm${}^{3}$. Up to the second pass was included in this range. As described above, not only is it difficult to make accurate predictions in this range, but also, the actual angular distortion is small. Figure 10c shows the experimental results and the predicted curves of the angular distortion. The error between experiments and calculations at the final welding pass was up to −7.7%, which indicated that the calculation agreed well with the experiments. These results reveal that the assumption that the deformation behavior of bead-on-plate welding is equivalent to that of the V-groove can be applied for distortion prediction. Final angles and errors are summed up in Table 4.

### 3.2. Effect of the Number of Welding Passes on Angular Distortion

As shown in Figure 10, the angular distortion was greatly influenced by the number of welding passes regardless of the amount of heat input. Furthermore, the angular distortion was very small in the first and second pass, but increased rapidly in the third to fifth pass, then gradually decreased with further passes. This implies that the angular distortion had the maximum at a specific thickness and heat input.

**Figure 11:**
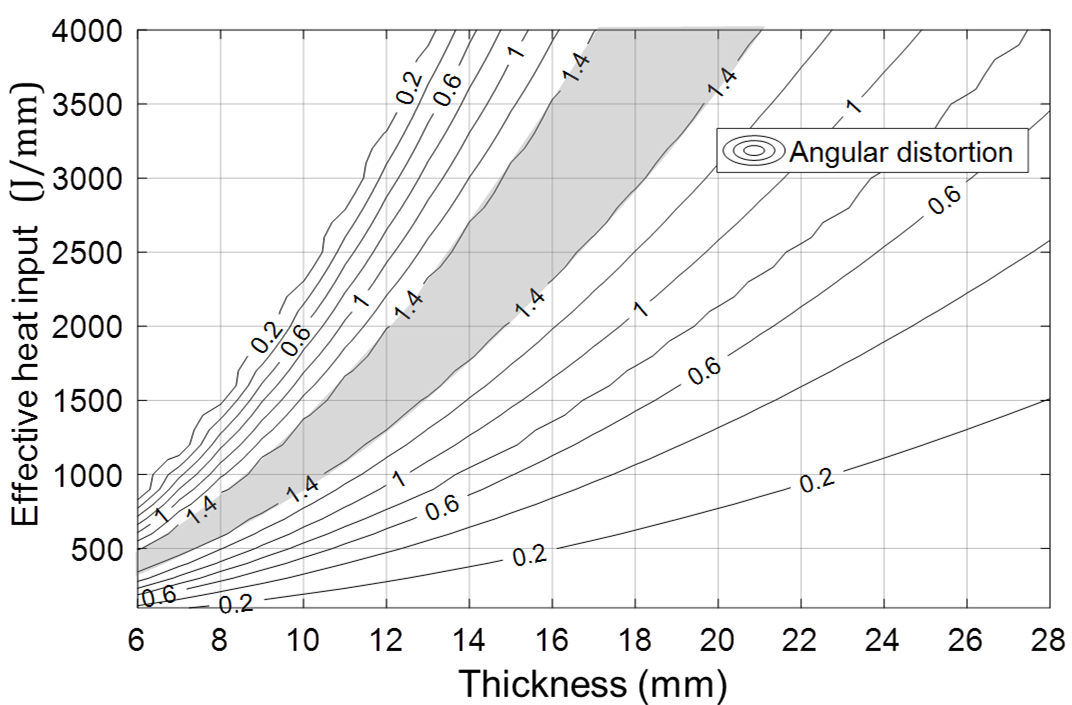
Contour of angular distortion with heat input and thickness; the gray zone shows the conditions for the maximum angular distortion.

Database #2 can be converted to the contour of the angular distortion as shown in Figure 11. The gray zone indicates the conditions for the maximum angular distortion. Most multi-layer welding can experience this zone if the heat input remains almost constant and the layer thickness increases equally. In the typical V-groove, this zone lies between the third to fifth pass during multi-layer welding. One way to minimize the distortion in multi-layer welding is to avoid this condition and reduce the number of passes in this zone.

### 3.3. Effect of Parent Material Thickness on Angular Distortion

**Figure 12:**
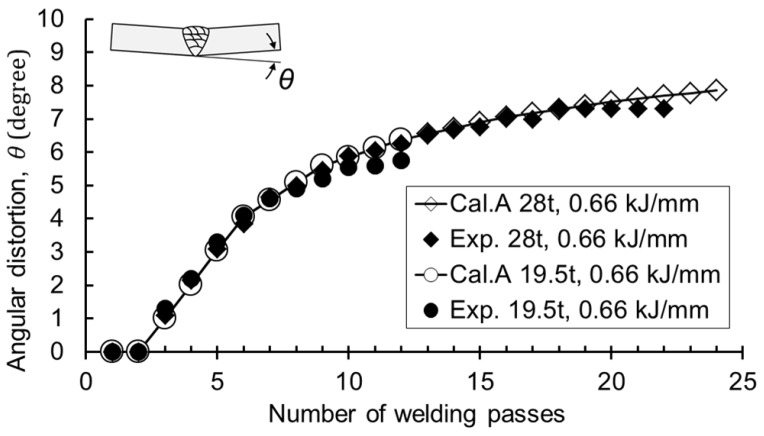
Results of angular distortion with different thicknesses.

Experiments and calculations were performed to investigate whether the thickness of the base material affected the deformation. The target thickness was 19.5 mm and 28 mm. The results exhibited that the angular distortion converged to one curve for both the experiment and calculation cases, as shown in Figure 12. However, the final angle was different depending on the thickness. Since the cross-sectional area of each bead was the same under the same heat input, the thicker base material had more welding passes, resulting in greater angular distortion at the final pass. Since the angular distortion was located on the prediction curve, the final angle was determined by the number of passes due to the increase in thickness.

### 3.4. Effect of Previous Weld Beads on Angular Distortion

**Figure 13:**
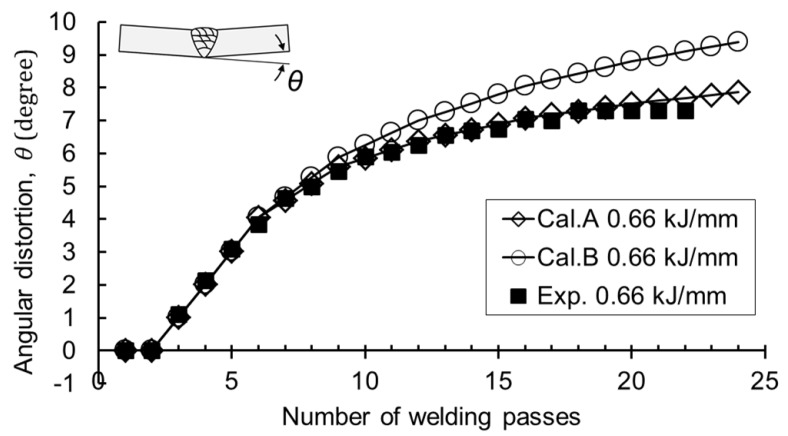
Comparison of the calculation; Cal.A: considering the previous bead, Cal.B: not considering the previous bead.

The proposed method also used the curve B of Figure 6b that did not take the previously-deposited bead into account. The calculation results using both the curve A and B are shown in Figure 13. The angular distortion by Curve B became greater than that by Curve A from about the tenth pass. The final angular distortion of the experiment and calculation by Curve A was 7.3 and 7.4 degrees, respectively, and prediction by Curve B was 9.4 degrees. The error at the final pass was almost 30%. The heat input-thickness parameter reached the bifurcation point from about the tenth pass of welding, where the gap between the curves A and B began to spread. When the parameter was less than 6 kJ/mm${}^{3}$, the calculation not considering the previous beads gave rise to larger angular distortion. As a result, the previous bead had a significant effect on the angular distortion, so it should be considered for predicting the angular distortion in actual multi-layer welding.

### 3.5. Effect of Bead Size on Angular Distortion

**Figure 14:**
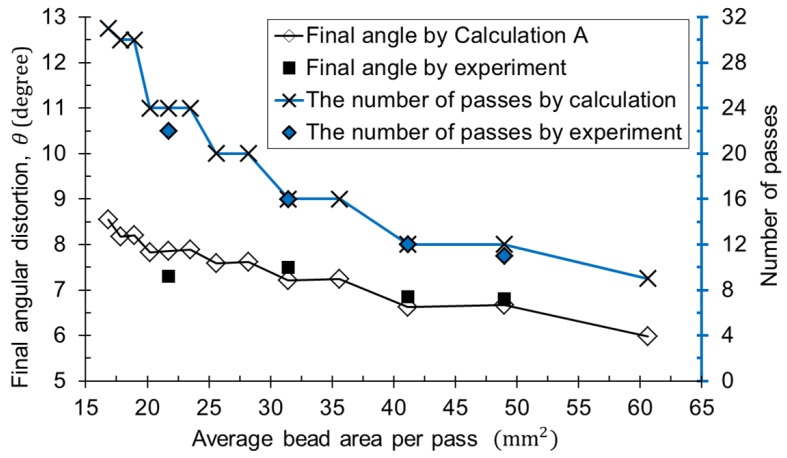
Final angular distortion with different bead areas per pass and the number of passes.

Experiments always have errors. On the other hand, algorithmic calculations are based on ideal assumptions, which can help to understand the physical phenomena of complex problems such as the prediction of the multi-layer welding. Figure 14 shows the angular distortion at the final welding pass with the cross-sectional area of the bead. The final angle decreased stepwise as the average bead area per pass increased if the shape of the weld joint was the same. However, there was a case where the bead area was different in the same number of passes. For example, the heat input of 1.8 and 1.3 kJ/mm with 12 passes produced the bead area of 48.95 and 41.17 mm${}^{2}$. The final angles were calculated to be 7.31 and 7.27 degrees, respectively. They can be considered as the same value. As a result, the final angular distortion decreased as the number of passes decreased under the same conditions. The smaller the number of passes, the greater the cross-sectional area per pass, but not vice versa. According to the job knowledge contents of TWI (The Welding Institute) [33] welding distortion can be minimized by welding with a small number of passes with a large bead size, which is consistent with the results of this study. It should be noted that this method initializes the bending angle up to the second pass, so actual angular distortion at the final pass may vary slightly.

### 3.6. Effect of Weld Joint Geometry on Angular Distortion

**Figure 15:**
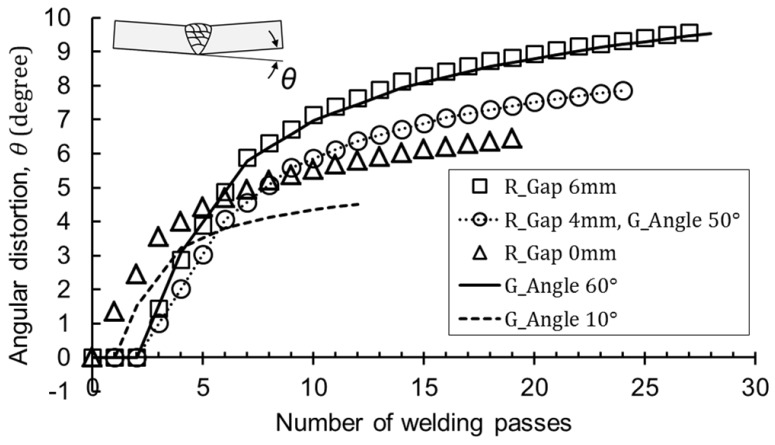
Calculation results with the root gap (R gap) and groove angle (G angle).

The algorithm was used to calculate the angular distortion about arbitrary joint shapes. Figure 15 shows that as the root gap or the groove angle increased, not only the final angular distortion, but also the magnitude of the angle per pass increased. As a result, the prediction curve depended on the shape of the weld joint, but not on the heat input or base metal thickness.

## 4. Conclusions

A method of predicting angular distortion in multi-layer welding has been developed using the relationship between welding conditions and geometrical information. Given the experimental data of bead-on-plate welding, it has proven possible to predict angular distortion in multi-layer butt welding using geometric principles. The algorithm procedure is summarized as follows.

- Information on the welding conditions and the weld joint geometry was the input.
- The bead area was calculated through Database #1, which consisted of the current-velocity parameter and bead size.
- The bead height was obtained from the geometric relationship between the bead area and the joint geometry, where the bead height plus previous thickness were used as the thickness in the next layer calculation.
- The angular distortion was estimated through Database #2, which is composed of the heat input-thickness parameters and the angular distortion.
- Accumulated angular distortions were obtained by iterative calculation of the above procedures.

The proposed methodology was verified through welding experiments on V-groove butt joints. From these results, the findings of the multi-layer welding distortion were as follows.

- The bead cross-sectional area was proportional to the square of the electric current divided by the welding speed, and the angular distortion was a function of the heat input divided by the square of the thickness.
- The effect of the previous layer on reduction in angular distortion should be applied to the prediction method.
- In the same weld joint, the angular distortion with the number of passes created a single curve regardless of the welding conditions, so the final angle was determined by the final pass number. The predictive curve, on the other hand, varied with the shape of the weld joint.
- There existed a region where the angular distortion was the maximum at a specific thickness and heat input. In the case of V-butt welding, the change in angular distortion was greatest in between the third and fifth welding pass.
- Reducing the number of passes with a large bead size decreased the welding angular distortion in the same joint.

## Figures and Tables

**Table 1:** Chemical compositions and mechanical properties of base and wire materials.

Type	Chemical Compositions (Mass %)				Mechanical Properties as Welded		
	C	Mn	Ni	Cr	Yield strength	Tensile strength	Elongation
LR-AH32 (base)	0.16	1.12	0.04	0.03	348 MPa	495 MPa	30%
E81T1-K2C (wire)	0.03	1.28	1.49	0.03	549 MPa	617 MPa	31%

**Table 2:** Experimental conditions for bead-on-plate welding.

Thickness (mm)	Voltage (V)	Current (A)	Speed (cm/min)	Number of Layers
11.5	29	285	15, 20, 22, 25	1, (6–13 passes per layer)
12	29	285	25, 30, 40, 60	4, (4–27 passes per layer)
15	29	285	25, 30, 40, 60	2, (6–13 passes per layer)
19.5	29	285	25, 30, 40, 60	2, (6–13 passes per layer)
28	29	285	25, 30	2, (6–13 passes per layer)
45	29	285	25, 30, 40, 60	2, (6–13 passes per layer)

**Table 3:** Welding conditions for V-groove butt welding.

Thickness (mm)	Voltage (V)	Current (A)	Speed (cm/min)	Effective Heat Input (kJ/mm)
19.5, 28	29	285	60, 40, 20, 25	0.7, 1.0, 1.3, 1.6

**Table 4:** Final angles by experiment and calculation.

Effective Heat Input (kJ/mm)	0.7	1.0	1.3	1.6
Experiment (degree), [Number of passes]	7.3, [22]	7.5, [16]	6.9, [12]	6.9, [11]
Calculation (degree), [Number of passes]	7.9, [24]	7.2, [16]	6.6, [12]	6.7, [12]
Error (%)	−7.7	3.9	3.2	3.3
Error (%) = (Experiment − Calculation)/Experiment × 100				

## Abbreviations

The following abbreviations are used in this manuscript:

HAZ	Heat-affected zone
FCAW	Flux cored arc welding
GMAW	Gas metal arc welding
SAW	Submerged arc welding
